# The Presence of MRZ Reactions Improves the Prediction of Multiple Sclerosis in Children with Optic Neuritis

**DOI:** 10.3390/children12040497

**Published:** 2025-04-13

**Authors:** Franziska Kauth, Sophie Chen, Eva-Maria Wendel, Markus Reindl, Nicole Heußinger, Kevin Rostásy

**Affiliations:** 1 Department of Pediatric Neurology, Children’s Hospital Datteln, University Witten/Herdecke, D-45711 Datteln, Germany; 2 Olgahospital Stuttgart, Kinderneurologie, D-70174 Stuttgart, Germany; 3 Clinical Department of Neurology Innsbruck, Medical University of Innsbruck, A-6020 Innsbruck, Austria; 4 Department of Pediatrics, Nuremberg General Hospital, Paracelsus Medical University, D-90471 Nuremberg, Germany

**Keywords:** pediatric onset multiple sclerosis, MRZ reaction, optic neuritis

## Abstract

**Background/Purpose:** Optic neuritis (ON) is a rare disease that may remain a single episode or transform into MS. OCBs and spinal MRI lesions have already been identified as prognostic factors. Aim: Our aim was to evaluate if the presence of more than one elevated antibody index of measles, rubella, and/or varicella-zoster (MRZ) is an indicator of risk of conversion. **Methods:** In total, 228 patients diagnosed with ON between 1990 and 2013 were included in this retrospective study. All children had a data set consisting of age, sex, ON type, MRI, and detailed CSF studies, including the presence of OCBs and MRZ reactions and a follow-up of at least 1.5 years. Children were then divided into two groups: those who developed MS according to the McDonald criteria 2010 (n = 92) and those who did not (n = 136). Binary logistic regression analysis was used to assess the relationship between the different prognostic factors and conversion to MS. Positive (PPV) and negative predictive values were calculated. **Results:** Binary logistic regression analysis revealed that an MS-like MRI (*p* < 0.001), positive OCBs (*p* = 0.002), and a positive MRZ reaction (*p* < 0.001) were significant prognostic factors for conversion to MS after ON. Calculated PPVs showed a positive MRZ reaction alone to already be a good predictor (PPV 0.90 (95%CI: 0.82 to 0.95), *p* < 0.001). The best prediction was possible with a combination of cMRI, the presence of OCBs, and a positive MRZ reaction (PPV 1.00 (0.93 to 1.00), *p* < 0.001). **Conclusions:** Our findings show that a positive MRZ reaction alone already has a high predictive value for future conversion to MS and should be included in the workup of a child with an initial demyelinating event.

## 1. Introduction

Optic neuritis (ON) is an inflammation of the optic nerve which can be an isolated event or part of a range of different acute demyelinating syndrome (ADSs) such as neuromyelitis optica spectrum disorder (NMOSD), MOG autoantibody-associated disorder (MOGAD) or multiple sclerosis (MS), in which it often presents as the first episode of the disease [1,2]. Several studies have already looked at biomarkers that are associated with further conversion to MS after the initial event [3,4]. Heussinger et al. found that children with ON who harbor oligoclonal bands (OCBs) have a markedly increased risk for further episodes and the diagnosis of MS. The authors further showed that the presence of additional OCBs in CSF and an MS-like MRI does increase the odds ratio for MS even further [2], leading to the inclusion in the McDonald criteria 2017 as a surrogate marker for dissemination in time (DIT) [5]. Another biomarker thought to be highly specific for MS—but less well studied in children in particular—is the so-called presence of a positive MRZ reaction, which is a poly-specific immune reaction in which there is an intrathecal synthesis of at least two or more antibody specificities, most frequently against measles (M), rubella (R) and varicella-zoster (Z) (MRZ) [6,7]. The MRZ reaction is seen as an expression of a long-lasting, autoimmunological inflammatory reaction, which is frequently seen in adult MS patients. The MRZ reaction as an early biomarker for MS has gained more attention recently in adults and will most likely be included in the upcoming revision of the McDonald criteria.

Therefore, our aim was to evaluate if a positive MRZ reaction could serve as a biomarker for the risk of conversion in children with an initial ADS. Secondly, we wanted to assess how sensitive the MRZ reaction is compared to already-known biomarkers, such as the presence of OCBs and MRI lesions.

## 2. Materials and Methods

**Patients:** We retrospectively included 228 patients from 21 different hospitals between 1990 and 2013 if they were diagnosed with ON between the ages of 2 and 17 years. The 2010 McDonald criteria were used for the diagnosis of MS, which relied primarily on clinical presentation as the main diagnostic criterion. If there were two or more clinical relapses, no further evidence was required. If only one clinical relapse was observed, additional evidence of dissemination in space (DIS) (via MRI) and dissemination in time (DIT) (via clinical relapses or new MRI lesions) was required to confirm the diagnosis. The majority of children were part of a previously reported study by Heussinger et al., who studied the predictive value of the presence of OCBs for the further development of MS. In total, 22 children with ON from the BIOMARKER study were added for the purpose of the study.

Children were only included with a full data set consisting of age, sex, MRI, the presence of OCBs, and MRZ reaction. In addition, the following parameters were collected: time to MS development, follow-up time (FU), ON type (unilateral, bilateral), disease-modifying therapy (DMT) (yes/no), and the testing of antibodies against myelin-oligodendrocyte-glycoproteins (MOGs abs) in 32/228 patients.

All patients displayed the typical clinical signs of isolated unilateral or bilateral optic neuritis (ON), including vision loss, impaired color vision, and eye pain during eye movement. Bilateral ON was defined as either the simultaneous onset of ON in both optic nerves or the occurrence of sequential ON in both eyes within a 4-week period. As part of the standard diagnostic evaluation, all patients underwent cerebral magnetic resonance imaging (cMRI) and testing for IgG OCBs in CSF. Additionally, all pediatric patients underwent supplementary laboratory testing to exclude other potential diagnoses, including neuroborreliosis. Abnormal cMRI findings, as determined by the neuropediatricians involved in the study, were defined as the presence of at least one lesion consistent with MS and a diameter greater than 3 mm located outside the optic nerves and chiasm. This retrospective study did not employ a standardized MRI protocol. A positive result for OCB was defined by the detection of at least two CSF bands that were absent from the serum. The analysis used was isoelectric, focusing on IgG immunoblotting or silver staining. (2). The assessment of the MRZR was performed with the analysis of virus-specific AIs against measles, rubella, and zoster and was defined as positive with an elevation of 2 or more indices with a value of more than 1.5. These were calculated as follows: AI = QIgG[spec]/QIgG[total] if QIgG[total] < Qlim and AI = QIgG[spec]/Qlim if QIgG[total] > Qlim according to Reiber’s formula [8,9]. In the second step, the cohort was divided into two different groups, depending on whether they developed MS or not.

**Statistical analysis**: SPSS version 29.0 and GraphPad Prism Version 10.4. were used. Binary logistic regression analysis was used to quantify the predictive strength of the different prognostic factors (age, sex, MRI, OCB, and MRZ) and conversion to MS and are shown with model fit, collinearity diagnostics, and calibration metrics. Positive (PPV) and negative predictive values (NPVs) with 95% confidence intervals (CI) were analyzed using Fisher’s exact test and the Wilson–Brown method for the calculation of 95% CIs. The resulting *p*-values were adjusted for multiple comparisons using Bonferroni’s correction.

**Standard Protocol Approvals, Registrations, and Patient Consent**: The study was conducted in accordance with the Declaration of Helsinki, and the protocol was approved by the Ethics Committee of Witten/Herdecke University, Germany (164/2014), on 10.10.2020.

## 3. Results

In total, 228 patients with ON were included, of whom 92 (40.53%) developed MS. The median age of onset was 14.3 years in the MS group and 12.1 years in the non-MS group. The gender distribution was equal in both groups. In total, 72.8% of children who developed MS and 75% of children who did not develop MS were female. The mean time to develop MS was 229 days (range 0 to 1278 days), and the mean FU time of children who developed MS was 3.44 years (range 1 to 7 years). The mean FU time in children who did not develop MS was 4.58 years (range 2 to 20 years). No children were lost during the follow-up period.

As can be seen in Table 1, of those children who developed MS, 72/92 children (78.3%) had positive OCBs in CSF, 87/92 (94.6%) had an abnormal MRI, and 73/92 (79.3%) a positive MRZ reaction. In the non-MS group, only 30/136 (22.1%) children had a positive OCBs, 25/136 (18.4%) children had an abnormal MRI, and 8/136 (5.9%) children had a positive MRZ reaction. All children were tested for MRZ reactions at baseline. In the MS group, 85/92 (92.4%) children had a unilateral and 7/92 (7.6%) children had bilateral optic neuritis; in the non-MS group, 102/136 (75%) children had a unilateral and 34/136 (25%) children had bilateral optic neuritis. In total, 88 of 92 children with MS received disease-modifying therapy. In total, 32/228 children were tested for MOG-abs. In total, 2/12 children who developed MS had positive MOG abs, but all had low titers and did not fulfill the MOGAD criteria, and 10/20 children did not develop MS but fulfilled the radiological criteria for MOGAD.

In Table 2, factors influencing the risk of conversion to MS after ON calculated by binary logistic regression analysis are shown. Binary logistic regression analysis revealed an MS-like MRI (*p* < 0.001), positive OCBs (*p* = 0.002), and a positive MRZ reaction (*p* < 0.001) to be significant prognostic factors for conversion to MS after ON, while age and sex were not reported (*p* = 0.533 and *p* = 0.355).

In the second step, positive and negative predictive values for the significant factors indicated by the binary regression analysis were calculated, as can be seen in Table 3. An MS-like MRI (PPV = 0.78, NPV = 0.96), positive OCBs (PPV = 0.71, NPV = 0.84), and a positive MRZ reaction (PPV = 0.90, NPV = 0.87) were already shown to be significant. The best prediction was possible with a combination of cMRI, the presence of OCBs, and a positive MRZ reaction (PPV 1.00 (95% CI 0.93 to 1.00), *p* < 0.001).

## 4. Discussion

In our study of 228 children with ON, we show that the presence of a positive MRZ reaction has a strong predictive value for conversion to MS in children with an initial episode of ON. We further show that the combination of two of the three factors mentioned above offers an even better prognosis, and the best prediction with a PPV of 1.0 was possible with a combination of all three factors, including positive OCBs in CSF, an abnormal/MS-like MRI, and a positive MRI reaction.

The MRZ reaction as a prognostic tool has already been studied in adults with the presence of a positive MRZ reaction being associated with an increased risk for conversion to relapse-remitting MS as well as for primary-progressive MS [10,11,12,13]. These studies also showed that the MRZ reaction has a high specificity, and that positive OCBs have a high sensitivity for conversion to MS [12,14,15], which underlines the usefulness of these biomarkers, in particular in those cases where patients have symptoms of a demyelinating disease such as MS but do not have positive OCBs [5,12,14,16,17]. Also, in our cohort, of 20 children, who were diagnosed with MS and who did not have positive OCBs, 16 children had a positive MRZ reaction. Interestingly, eight children with a positive MRZ reaction did not develop MS. The mean FU in this group was 3.83 years (ranging from 2 to 5.83 years). An explanation might be that the MRZ reaction is a result of a non-specific bystander activation of B cells or due to molecular mimicry that may be associated with autoimmunity but may not actually be the cause of disease onset.

In addition, a positive MRZ reaction has been shown to be less prevalent in almost all patients with other pathogen-driven inflammatory or autoimmune-mediated CNSs such as Lyme neuroborreliosis HTLV-1-associated myelopathy or MOGAD [5,10,18,19,20].

Little is known about the frequency of a positive MRZ reaction in larger cohorts of children with MS. In a recent study, Chen et al. showed that a positive MRZ response can be found in every fourth child at the time of MS diagnosis [7]. Even if there was no evidence that children with a positive MRZ reaction also have a worse disease course, it is still helpful for the diagnosis. As it has been shown to not only be a “rule-in” marker, the MRZ reaction qualifies for providing a conclusive and affirmative diagnostic in comparison to OCBs and IgG, which have been seen as “rule-out” markers [7,21]. Another conclusive marker seems to be the central vein sign (CVS), an MRI-detectable central vein inside white matter lesions, as it is said to be able to differentiate MS from other diseases [22].

As in the study by Heussinger et al. [2], we discovered a conversion rate to MS of 40%, which is much higher than those reported in several studies before, where a conversion rate between 0 and 30% was reported [23]. We, therefore, suggest including the MRZ reaction in the upcoming revision of the MacDonald criteria in 2025 in addition to other markers such as the CVS in order to enhance the timely diagnosis of MS and prompt the initiation of DMT.

The immunological mechanisms leading to the MRZ reaction and the physiological role remain unclear. Poly-specific B-cell responses such as the bystander reaction and humoral immune mechanisms seem to play a role, but molecular mimicry and the potential for latent viral reactivation may also have an impact [12,24,25,26]. Nevertheless, further research is needed to understand more precisely how this works and in which areas the MRZ reaction might be helpful as a diagnostic tool.

Several limitations should be acknowledged. Firstly, the retrospective design of the study may introduce biases in the results. Secondly, for the diagnosis of MS, the 2010 McDonald criteria had to be applied because the data of a large majority of children in our cohort were collected before the 2017 criteria were established. We are aware of the fact that if we had used the 2017 McDonald criteria, most likely, a proportion of our cohort would have already been diagnosed with MS at the initial event, particularly those showing the presence of OCBs and MRI findings indicating DIS. Additionally, certain data collection procedures, such as the MRI evaluations and follow-up assessments, were conducted by the pediatric neurologists at the respective clinics, which could have led to variability in the data acquisition. These limitations may have introduced a spectrum bias. Lastly, only a small subset of serum MOGAD analysis was performed, which may undermine the generalizability of the results.

## 5. Conclusions

The MRZ reaction is a powerful tool with a strong predictive value for conversion to MS in children after isolated optic neuritis. The best prediction was possible with a combination of positive OCBs, an abnormal MRI, and a positive MRZ reaction.

## 6. Future Direction

A comparison with emerging biomarkers (such as GFAP, NfL) and MRI markers (e.g., the central vein sign) might be very interesting. Additionally, an assessment of all children for MOGAD would be desirable. Furthermore, it remains important to examine why children with a positive MRZ reaction do not develop MS and whether they may still be at risk of developing the condition in the future.

## Figures and Tables

**Table 1:** Summary of clinical and demographic features of 228 children with optic neuritis grouped by development of multiple sclerosis.

Characteristics	MS	No MS	Total
Patients	92	136	228
Mean age in years	14.3 (SD 2.8)	12.1 (SD 3.4)	-
Sex Male Female	25 (27.2%) 67 (72.8%)	34 (25%) 102 (75%)	59 169
Mean time to MS development in days	229	-	-
OCB positive Yes no	72 (78.3%) 20 (21.7%)	30 (22.1%) 106 (77.9%)	102 126
MRI abnormal Yes no	87 (94.6%) 5 (5.4%)	25 (18.4%) 111 (81.6%)	112 116
MRZ reaction Positive negative	73 (79.3%) 19 (20.7%)	8 (5.9%) 128 (94.1%)	81 147
ON type Unilateral bilateral	85 (92.4%) 7 (7.6%)	102 (75%) 34 (25%)	187 41
Mean FU time in years	3.44	4.58	-
Immunomodulatory therapy yes no	88 (95.7%) 4 (4.3%)	8 (5.9%) 128 (94.1%)	96 132
MOG abs Yes no	2 (16.7%) 10 (83.3%)	10 (50%) 10 (50%)	12 20

**Table 2:** Factors influencing the risk of conversion to multiple sclerosis after isolated optic neuritis: results of binary regression analysis.

	Non-MS (136)	MS (92)	Exp(B) 95% CI	*p*-Value *
Age (years)	12.7 (4.3–17.9)	14.7 (5.0–17.9)	1.07 (0.87 to 1.30)	0.533
Sex (female)	102 (75%)	67 (73%)	1.87 (0.50 to 7.04)	0.355
MRI (MS-like)	25 (18%)	87 (95%)	146.01 (15.96 to 1335.82)	<0.001
OCB (pos)	30 (22%)	72 (78%)	7.10 (2.05 to 24.64)	0.002
MRZ (pos)	8 (6%)	73 (79%)	156.64 (19.13 to 1282.90)	<0.001

* Model fit: Chi-square = 233.59, df = 5, *p* < 0.001; Cox and Snell R Square = 0.641, Nagelkerke R Square = 0.866. Collinearity Statistics (VIF, tolerance): Age: 1.09 (0.92), cMRI 2.49 (0.40), OCB 1.07 (0.94), MRZ 2.48 (0.40); Sex: 1.04 (0.96); Hosmer and Lemeshow Test: Chi-square 0.977, df = 8, *p* = 0.998.

**Table 3:** Positive and negative predictive values (PPV and NPV) calculated for significant prediction factors of the binary regression analysis.

	Non-MS (n = 136)	MS (n = 92)	PPV (95% CI)	NPV (95% CI)	*p*-Value *
cMRI (MS-like)	25 (18%)	87 (95%	0.78 (0.69 to 0.84)	0.96 (0.90 to 0.98)	<0.001
OCB (pos)	30 (22%)	72 (78%)	0.71 (0.61 to 0.79)	0.84 (0.77 to 0.89)	<0.001
MRZ (pos)	8 (6%)	73 (79%)	0.90 (0.82 to 0.95)	0.87 (0.81 to 0.92)	<0.001
cMRI + OCB	10 (7%)	68 (74%)	0.87 (0.78 to 0.93)	0.84 (0.77 to 0.89)	<0.001
cMRI + MRZ	1 (1%)	68 (74%)	0.99 (0.92 to 0.99)	0.84 (0.79 to 0.90)	<0.001
OCB + MRZ	2 (1%)	57 (62%)	0.97 (0.88 to 0.99)	0.79 (0.73 to 0.85)	<0.001
cMRI + OCB + MRZ	0 (0%)	53 (58%)	1.00 (0.93 to 1.00)	0.78 (0.71 to 0.83)	<0.001

* Positive and negative predictive values were calculated using Fisher’s exact test and the Wilson–Brown method. *p*-values were adjusted for seven comparisons using Bonferroni’s correction.

## Data Availability

The data presented in this study are available on request from the corresponding author due to ethical reasons.

## Abbreviations

The following abbreviations are used in this manuscript:

AAb	autoantibody
Abs	autoantibodies
ADS	acute demyelinating syndrome
CI	confidence interval
CSF	cerebrospinal fluid
CVS	central vein sign
DIS	dissemination in space
DIT	dissemination in time
FU	follow-up
MOGAD	MOG autoantibody-associated disorder
MRI	magnetic resonance imaging
MRZ	measles, rubella, and zoster
MS	multiple sclerosis
NPV	negative predictive value
NMOSD	neuromyelitis optica spectrum disorder
OCBs	oligoclonal bands
ON	optic neuritis
PPV	positive predictive value
SD	standard deviation
